# Supplementary material for: Proline to Threonine Mutation at Position 162 of NS5B of Classical Swine Fever Virus Vaccine C Strain Promoted Genome Replication and Infectious Virus Production by Facilitating Initiation of RNA Synthesis
Source: Viruses. 2021 Aug 2;13(8):1523. doi: 10.3390/v13081523 (PMC8402891; doi:10.3390/v13081523)
Supplement: Supplementary file 1 [file viruses-13-01523-s001.zip › viruses-1236941-supplementary.pdf]

Supplementary Materials:

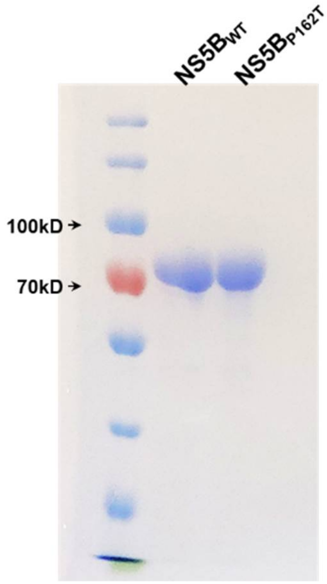

Figure S1. SDS-PAGE gel of the purified NS5B$_{WT}$ and NS5BP$_{162T}$. Wild-type and mutant NS5B proteins were purified to near homogeneity as judged by SDS-PAGE.

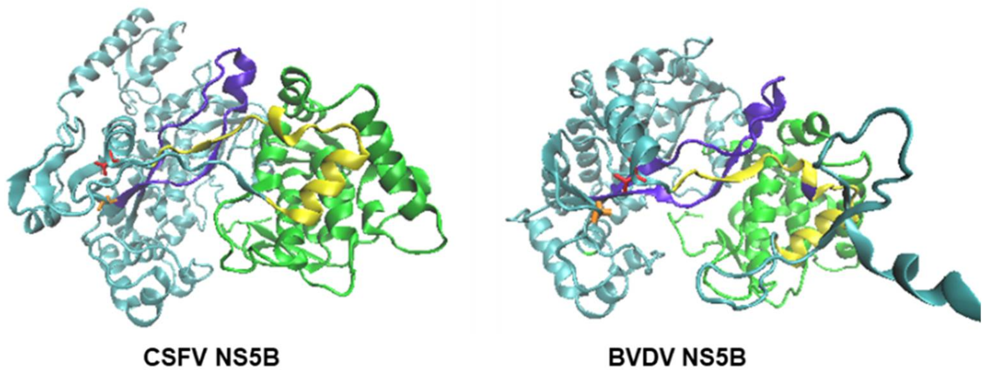

Figure S2. Relative positions of residue 162 and residue 392 in CSFV and BVDV NS5B confirmation displayed by VMD software based on previous reports [31, 35]. Residue 162 (red) and residue392 (orange) were marked in the structures. The fingertip region contains the N-terminal part of the finger domain (yellow) and the insertion in the β-finger domain (violet). The fingertip bridges the finger domain and thumb domain (green). Residue 162 is located at the C-terminal of one part (yellow) of the fingertip region, and residue 392 is located at the tip of the middle finger close to residue 162. The accession codes of CSFV and BVDV NS5B in the Protein Data Bank respectively are 5Y6R and 1S48 [31, 35].

Table S1. Analysis of two CSFV C strains based on amino acid sequences

Vrial protein	Amino acid position in polyprotein	C strain	C/HVRI strain
E0	446	S	A
E1	618	V	I
E2	1053	A	V
p7	1122	T	A
NS2	1156	V	I
	1317	V	I
NS5A	2980	T	A
	3005	K	N
NS5B	3818	M	I
